# Nanogel Loaded with *Perilla frutescens* Leaf-Derived Exosome-like Nanovesicles and Indomethacin for the Treatment of Inflammatory Arthritis

**DOI:** 10.3390/biology14080970

**Published:** 2025-08-01

**Authors:** Xianqiang Li, Fei Wang, Rui Wang, Yanjie Cheng, Jinhuan Liu, Wanhe Luo

**Affiliations:** 1 College of Biomedicine and Health, Anhui Science and Technology University, Chuzhou 233100, China; lixq@ahstu.edu.cn (X.L.); 19105527543@163.com (F.W.); wangrui@ahstu.edu.cn (R.W.); chengyj@ahstu.edu.cn (Y.C.); 2 College of Animal Science and Technology, Tarim University, Alar 843300, China; liujinhuan0830@163.com

**Keywords:** inflammatory arthritis, *Perilla frutescens* leaves, exosome-like nanovesicles, nanogel, topical drug delivery systems

## Abstract

Inflammatory arthritis (IA) is a chronic condition marked by joint dysfunction and pain, posing significant challenges for effective drug delivery. This study separated *Perilla frutescens* leaf-derived exosome-like nanovesicles (PFE) to effectively penetrate the stratum corneum barrier. These nanovesicles and indomethacin (IND) were subsequently developed into a nanogel designed for topical drug delivery systems (PFE-IND-GEL). PFE exhibited a typical vesicular structure with a mean diameter of 98.4 ± 1.3 nm. The hydrodynamic size and zeta potential of PFE-IND-GEL were 129.6 ± 5.9 nm and −17.4 ± 1.9 mV, respectively. Mechanistic investigations in HaCaT keratinocytes showed that PFE significantly downregulated tight junction proteins (ZO-1 and Occludin, *p* < 0.01) via modulation of the IL-17 signaling pathway, as evidenced by transcriptomic analysis. In a sodium urea crystal-induced rat IA model, the topical application of PFE-IND-GEL significantly reduced joint swelling (*p* < 0.05) and serum levels of inflammatory cytokines (IL-6, IL-1α, TNF-α) compared to control groups. Histopathological analysis confirmed the marked attenuation of synovial inflammation and cartilage preservation in treated animals. These findings underscore the dual role of PFE as both a topical permeation enhancer and an anti-inflammatory agent, presenting a promising strategy for managing IA.

## Simple Summary

Indomethacin continues to be a first-line therapeutic agent for various inflammatory conditions, including inflammatory arthritis. However, its conventional oral tablet formulations are associated with suboptimal anti-inflammatory efficacy and gastrointestinal adverse effects. Our multi-omics investigations at both genomic and proteomic levels have revealed that *Perilla frutescens* leaf-derived exosome-like nanovesicles can attenuate the stratum corneum barrier by downregulating tight junction protein expression in keratinocytes through modulation of the IL-17 signaling pathway. Leveraging this mechanism, this work engineered a topical application of indomethacin-*Perilla frutescens* leaf-derived exosome-like nanovesicle nanogel formulation. And in vivo pharmacodynamic evaluations demonstrated a significant anti-arthritic efficacy of this innovative delivery system. These findings position *Perilla frutescens*-derived extracellular vesicles as promising biological enhancers capable of overcoming epidermal barrier limitations. Therefore, the extracellular vesicles derived from *Perilla frutescens* leaves may have promising application potential in the development of topical preparations to overcome the barrier of the stratum corneum.

## 1. Introduction

Inflammatory arthritis (IA) is a chronic disease characterized by limited mobility, joint destruction, pain, and swelling that severely limits the patient’s activities of daily life [1]. Additionally, it is common for people with IA to have a low ability to work, and they also face a high risk of losing their jobs and being permanently excluded from the labor market [2]. The annual prevalence of IA in general practice was 4.8 per 1000 patients in the Netherlands [3] and more than 50,000 people have been diagnosed in Denmark [2]. More seriously, it is reported that the global prevalence of IA is increasing and shows a younger trend in athletes and obese people [4].

Although scientific research data have suggested that micronutrients [5] and nanomaterials [6] can be used as a cure for IA, nonsteroidal anti-inflammatory drugs (NSAIDs) are commonly used clinically to combat inflammation and to effectively relieve IA symptoms [7]. Among NSAIDs, indomethacin (IND) is the first drug that can inhibit prostaglandin synthesis produced by cyclooxygenase enzymes, which are critical mediators of IA [8]. However, IND oral administration is plagued by gastrointestinal complications and suboptimal joint bioavailability due to impaired blood perfusion in inflamed tissues [9]. While topical drug delivery systems (TDDS) offer a promising alternative to circumvent hepatic first-pass metabolism and enable sustained release, the formidable stratum corneum barrier—particularly its corneocyte–lipid matrix and tight junction complexes—severely limits drug permeation [10].

Recent advances in extracellular vesicle research have unveiled plant-derived exosomes as novel biocompatible carriers capable of modulating skin permeability [11]. *Perilla frutescens*, a traditional medicinal herb, contains bioactive constituents (e.g., rosmarinic acid, perillaldehyde, galactoglycerolipids [12]) with documented anti-inflammatory and antioxidant properties [13]. Crucially, its exosomal fraction has shown unique capacity to regulate epithelial barrier function through intercellular communication mechanisms [14]. Accordingly, we hypothesize that *Perilla frutescens*-derived exosomes (PFE) could synergistically enhance topical NSAID delivery for the treatment of IA by dual mechanisms: (1) transiently downregulating keratinocyte tight junction proteins to bypass stratum corneum barriers, and (2) exerting intrinsic anti-inflammatory effects to potentiate drug efficacy.

In this study, we leveraged the UV-responsive properties of the photoinitiator (LAP) and the adhesion characteristics of Gelatin Methacryloyl (GelMA) and oxidized alginate to develop a nanogel aimed at enhancing its adhesion to joint skin and achieving localized drug retention. Initially, we employed ultracentrifugation combined with density gradient centrifugation to isolate and purify PFE [15]. Subsequently, we added PFE and IND to the nanogel system to prepare PFE-GEL and IND-GEL. In our mechanistic investigation, we concentrated on assessing the impact of PFE on the expression of tight junction proteins in HaCaT cells. Finally, to evaluate whether the prepared PFE-IND-GEL could enhance the therapeutic efficacy of IND for IA, we established a rat model induced by the injection of sodium urate crystal suspensions into the ankle joint and administered treatment accordingly. Pharmacodynamic studies were conducted utilizing various methods, including assessments of local joint swelling, measurement of inflammatory factors, and analysis through pathological sections.

## 2. Materials and Methods

### 2.1. Cell Culture and Animals

HaCaT human keratinocytes (CCTCC, Cat# SCSP-509) were cultured in Dulbecco’s Modified Eagle Medium/Nutrient Mixture F-12 (DMEM/F12, Gibco, 11330032, Grand Island, NY, USA) supplemented with 10% fetal bovine serum and 1% penicillin-streptomycin. Cells were maintained at 37 °C in a humidified 5% CO_2_ atmosphere and passaged at 80–90% confluence using 0.25% trypsin-EDTA solution. Cells were seeded at 1 × 10^5^ cells/well in 6-well plates and cultured for 48 h until 80% confluence prior to treatment.

SD rats (270–300 g) were purchased from Hefei Qingyuan Biotechnology Co., Ltd., Hefei, China, and were housed in the animal laboratory of Anhui Science and Technology University. All animals were fed antibacterial-free food and water and acclimatized in the animal facility for one week. The use of animals and all the experimental protocols concerning the handling of mice either complied with the ARRIVE guideline or were carried out following the National Research Council’s Guide for the Care and Use of Laboratory Animals, Animal Science Academy of Tarim University’s Ethics Committee (approved number: 2023008, date of approval 10 March 2023).

### 2.2. Isolation and Characterization of PFE

Fresh Perilla frutescens leaves (500 g) were homogenized in cold PBS (pH 7.4) containing protease inhibitor cocktail (Roche, 4693159001) using a BioGen Pro200 homogenizer (Pro Scientific, Oxford, CT, USA) at 15,000 rpm for 3 cycles (30 s pulse/30 s rest). Sequential centrifugation was performed at 300× *g* (10 min), 2000× *g* (20 min), and 10,000× *g* (30 min) at 4 °C. The supernatant was ultracentrifuged at 100,000× *g* for 70 min (Optima XE-100, Beckman Coulter, Brea, CA, USA) and purified using the VesPura™ Plant Exosome Isolation Kit (PrimCyt, Foshan, China, NV0001A) through density gradient centrifugation. The BCA kit (Beyotime Biotechnology, Shanghai, China) was used to analyze the protein concentration of PFE. The size of PFE was measured after a 100-fold dilution with distilled water using dynamic light scattering (ZS90, Malvern Instruments, Malvern, UK), and its shape was analyzed with a transmission electron microscope (TEM, Hitachi, JEM-1200EX, Tokyo, Japan).

### 2.3. Preparation and Characterization of PFE-GEL, IND-GEL, and PFE-IND-GEL

Sodium alginate (Sigma, Kawasaki, Japan, 180947) was oxidized in the dark using a solution of 10 mM sodium periodate (Sigma, 311448) for a duration of 6 h. For the formation of nanogels, add 3.75 mL of a 5% (*w*/*v*) oxidized alginate to a round-bottom flask, followed by the addition of 10 mL of deionized water. After stirring for 30 min, incorporate 5 mL of a 10% (*w*/*v*) GelMA solution (Suzhou Intelligent Manufacturing Research Institute, Suzhou, China), along with 0.25 mL of a 50% (*w*/*v*) LAP solution (Suzhou Intelligent Manufacturing Research Institute, Suzhou, China) and 5 mL of a 10% CaCl_2_ solution. Continue stirring while gradually adding PFE (1 mL), IND (1 g), and PFE (1 mL) plus IND (1 g) dropwise to the mixture, (Dingyuan Biotechnology Co., Ltd, Alar, China), resulting in the formation of PFE-GEL, IND-GEL, and PFE-IND-GEL. The exact composition of the GEL is shown in Table 1.

The hydrodynamic diameters and zeta potentials of PFE-GEL, IND-GEL, and PFE-IND-GEL were measured after a 100-fold dilution with distilled water using the dynamic light scattering method (ZS90, Malvern Instruments, Malvern, UK). After freeze-drying PFE-GEL, IND-GEL, and PFE-IND-GEL, the internal appearance of the nanogels was analyzed using SEM (APREO, Thermo Scientific Inc., Waltham, MA, USA). The changes in modulus (elastic modulus and viscous modulus) of the gel over time under stress were determined using a HAAKE MARS RS6000 rheometer (Thermo Scientific, Bremen, Germany). The appearance of the nanogel was captured using a regular camera.

### 2.4. Quantitative Real-Time PCR (qPCR)

HaCaT cells treated with 20, 50, and 100 μg/mL PFE (Protein concentration) for 24 h were subjected to RNA extraction. Total RNA was extracted with TRIzol reagent (Invitrogen, Waltham, MA, USA, 15596026). cDNA synthesis employed PrimeScript RT Master Mix (Takara, Kusatsu, Japan, RR036A). qPCR reactions were performed on a QuantStudio 5 system (Applied Biosystems, Waltham, MA, USA) using SYBR Green Master Mix (Takara, RR420A). Primer sequences are provided in Table 2.

### 2.5. Immunofluorescence Staining

Cells treated with 20, 50, and 100 μg/mL PFE (Protein concentration) for 24 h and fixed with 4% paraformaldehyde were permeabilized with 0.1% Triton X-100 and blocked with 5% BSA. Primary antibodies against ZO-1 (Invitrogen, 40–2200) and Occludin (Abcam, Cambridge, UK, ab167161) were applied overnight at 4 °C. Alexa Fluor 594-conjugated secondary antibodies (Invitrogen, A-11012) were used for detection. Nuclei were counterstained with DAPI (Sigma, D9542). Images were captured using a confocal microscope (LSM 880, Zeiss, Oberkochen, Germany).

### 2.6. Transcriptomic Analysis

After the cells were collected by the above-mentioned method, they were immediately frozen in a −80 °C refrigerator. The total RNA of large cells was isolated and purified using TRIzol RNA extraction reagent (Invitrogen, Carlsbad, CA, USA) in accordance with the manufacturer’s procedure. The construction of the cDNA library, sequencing and transcriptome data analysis were conducted by LC-Bio Technology Co., Ltd. (Lianchuan Bio, Hangzhou, China). The differentially expressed mRNAs were selected with fold change >2 or fold change <0.5 and with parametric F-test comparing nested linear models (*p* value < 0.05) by R package (version 4.5) edgeR (https://bioconductor.org/packages/release/bioc/html/edgeR.html, accessed on 23 November 2024).

### 2.7. Rat IA Model and Treatment

Add 1 g of urea and 1 g of sodium hydroxide to 100 mL of distilled water. Heat the mixture in an 80 °C water bath for 20 min, then cool to 4 °C to obtain sodium urate crystals. Subsequently, disperse these crystals in a solution of 10% Tween 80 in physiological saline to obtain a suspension of urea sodium crystals. Next, inject this suspension into the medial side of the ankle joint of an ether-anesthetized SD rat. Finally, pathological section images confirmed the successful establishment of IA.

IA model rats were randomly allocated into five therapeutic groups (*n* = 8) receiving daily topical applications (8:00 AM ± 1 h) for 7 consecutive days. The blank control group received no treatment, while the negative control group was administered the blank nanogel. The three treatment groups consisted of PFE-GEL, IND-GEL, and PFE-IND-GEL. To enhance topical drug absorption, hair at the ankle joint of the rats was carefully trimmed one day prior to administration. A volume of 1 mL of the nanogel, IND: 40% (*w*/*v*), was applied to medical gauze and irradiated with a UV lamp for 2 min before being swiftly transferred to the rat’s joint and secured in place. After a 7-day treatment period, we assessed both the swelling rate of the rat’s ankle joint and the levels of inflammatory factors in serum using ELISA kits. Furthermore, pathological sections were employed to evaluate the inflammatory status at the rat’s ankle joint.

### 2.8. Inflammatory Cytokine Measurement

Serum levels of IL-6 (Abcam, ab234570), IL-1α (R&D Systems, Minneapolis, MN, USA, DY400), and TNF-α (Invitrogen, BMS622) were quantified using commercial ELISA kits according to manufacturers’ protocols. Absorbance was measured at 450 nm on a microplate reader (BioTek Synergy H1, Agilent, Beijing, China).

### 2.9. Histopathological Analysis

Joint tissues were decalcified in 10% EDTA (pH 7.4) for 21 days and embedded in paraffin. Sagittal sections (5 μm) were stained with hematoxylin/eosin (H&E) and Safranin O-Fast Green. Histopathological changes were assessed using an optical microscope.

### 2.10. Statistical Analysis

Data are expressed as mean ± SD (*n* = 6). Multiple comparisons used one-way ANOVA with Tukey’s post hoc test (GraphPad Prism 9.0). *p* < 0.05 was considered statistically significant.

## 3. Results

### 3.1. Characterization of PFE, PFE-GEL, IND-GEL, and PFE-IND-GEL

**Figure 1:**
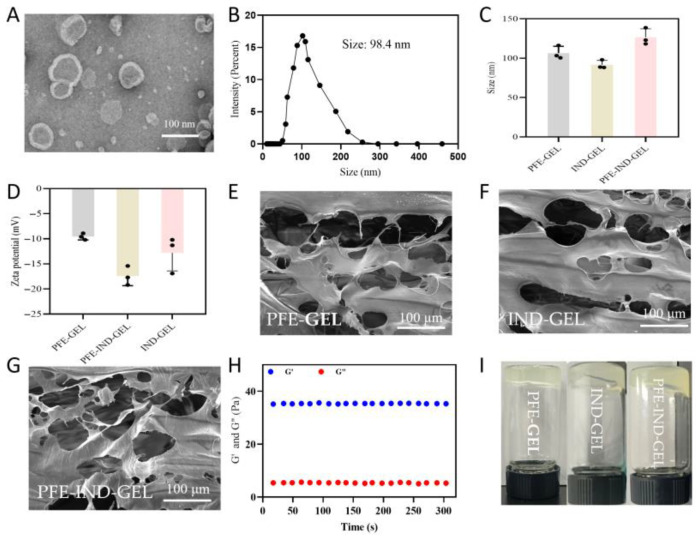
Characterization of PFE, PFE-GEL, IND-GEL, and PFE-IND-GEL. (**A**,**B**): TEM image and size distribution of PFE. (**C**,**D**): Size distribution and potential distribution of PFE-GEL, IND-GEL, and PFE-IND-GEL. (**E**–**G**): SEM image of PFE-GEL, IND-GEL, and PFE-IND-GEL. (**H**): Rheology of the nanogel. (**I**): Appearance of the nanogel.

TEM revealed that PFE exhibited a typical vesicular structure with intact bilayered membranes (Figure 1A). NTA demonstrated a homogeneous size distribution with a mean hydrodynamic diameter of 98.4 ± 1.3 nm (Figure 1B). The protein concentration of the three batches of purified PFE was determined using the BCA kit. The measurement result was that the protein concentration was 2.8 ± 0.2 mg/mL. PFE-IND-GEL showed a significantly larger particle size (129.6 ± 5.9 nm) compared to PFE-GEL (106.7 ± 6.9 nm) and IND-GEL (91.7 ± 5.5 nm) (Figure 1C). Zeta potential measurements indicated a surface charge of −17.4 ± 1.9 mV for PFE-IND-GEL, whereas PFE-GEL and IND-GEL exhibited a less negative charge (−9.6 ± 0.6 mV and −12.8 ± 3.6 mV) (Figure 1D). The freeze-dried PFE-GEL (Figure 1E), IND-GEL (Figure 1F), and PFE-IND-GEL (Figure 1G) exhibited a three-dimensional network structure, which significantly enhanced the efficacy of drug transportation. Rheological analysis revealed a frequency-dependent viscoelastic behavior, with storage modulus (G′) consistently higher than loss modulus (G″) within the 0.1–1000 Pa stress range (Figure 1H). And macroscopic evaluation showed stable gel formation at room temperature (Figure 1I).

### 3.2. PFE Reduces Tight Junction Protein Expression in HaCaT Cells

**Figure 2:**
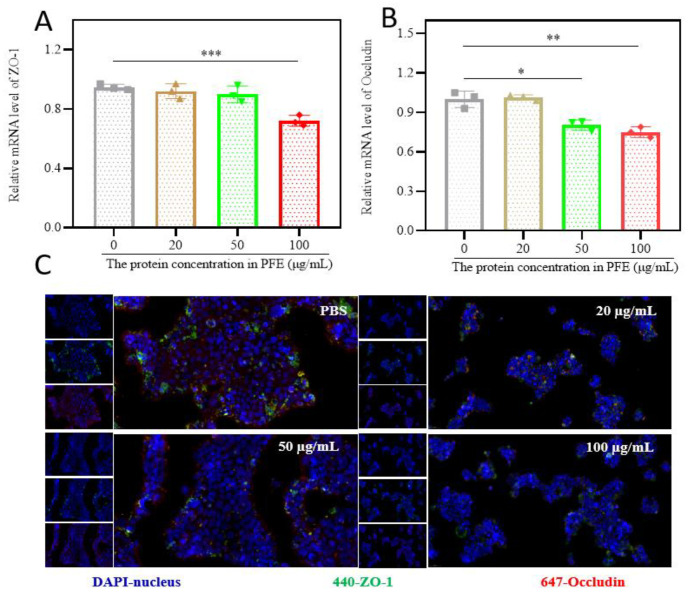
The effect of PFE on the expression of tight junction proteins in HaCaT cells. (**A**,**B**): The mRNA level of ZO-1 and Occludin. (**C**): Immunofluorescence confocal micrograph. * *p* < 0.05; ** *p* < 0.01; and *** *p* < 0.001.

Quantitative PCR analysis showed that PFE treatment significantly downregulated mRNA levels of ZO-1 and Occludin in HaCaT cells (Figure 2A,B). Immunofluorescence analyses further confirmed the reduced protein expression of ZO-1 and Occludin following PFE intervention (Figure 2C). These results suggest that PFE modulates epidermal barrier function by suppressing tight junction-related proteins.

### 3.3. Transcriptomic Profiling of PFE-Treated HaCaT Cells

**Figure 3:**
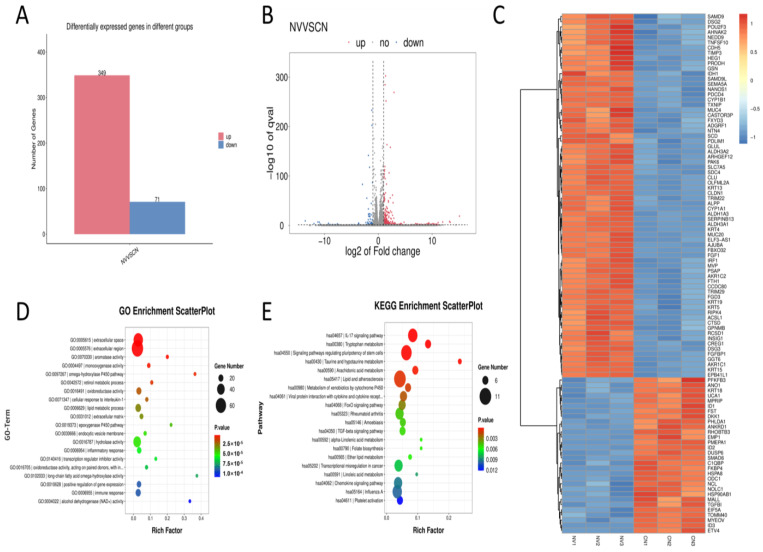
Transcriptional profiling analysis of PFE treatment on HaCaT cells. (**A**): Transcriptome sequencing revealed 420 differentially expressed genes (DEGs), 349 upregulated and 71 downregulated. (**B**): Volcano plot highlights the symmetrical distribution of upregulated (red) and downregulated (blue) genes at the log 2 FC threshold. (**C**): Clustering diagram of differentially expressed genes (red indicates high expression genes, blue indicates low expression genes). (**D**): GO functional enrichment analysis of differentially expressed genes. (**E**): KEGG pathway enrichment analysis of differentially expressed genes.

Transcriptome sequencing analysis (Figure 3A) identified a total of 420 differentially expressed genes (DEGs), of which 349 genes were significantly up-regulated and 71 genes were significantly down-regulated. Differential gene cluster analysis (Figure 3B) further verified the symmetrical distribution characteristics of differential genes under the log2 FC threshold, and the up-regulated genes (red) were statistically significantly different from the down-regulated genes (blue). The GO functional enrichment analysis graph (Figure 3C) showed that highly expressed genes (red) and low-expressed genes (blue) presented obvious expression pattern grouping. The volcano plot (Figure 3D) reveals the main functional categories of the differentially expressed genes in biological processes, molecular functions, and cellular components. KEGG pathway enrichment analysis (Figure 3E) showed that the differentially expressed genes were significantly enriched in key pathways such as IL-17 signaling pathway (hsa04657, gene number 11), tryptophan metabolism (hsa00380), and FoxO signaling pathway (hsa04068). Among them, the IL-17 signaling pathway had the highest enrichment factors, suggesting that this pathway had a central regulatory role in the study. In addition, arachidonic acid metabolism (hsa00590), rheumatoid arthritis (hsa05323) and other pathways were also significantly enriched, which may be closely related to phenotypic regulation.

### 3.4. Therapeutic Efficacy of PFE-IND-GEL in Arthritic Rats

**Figure 4:**
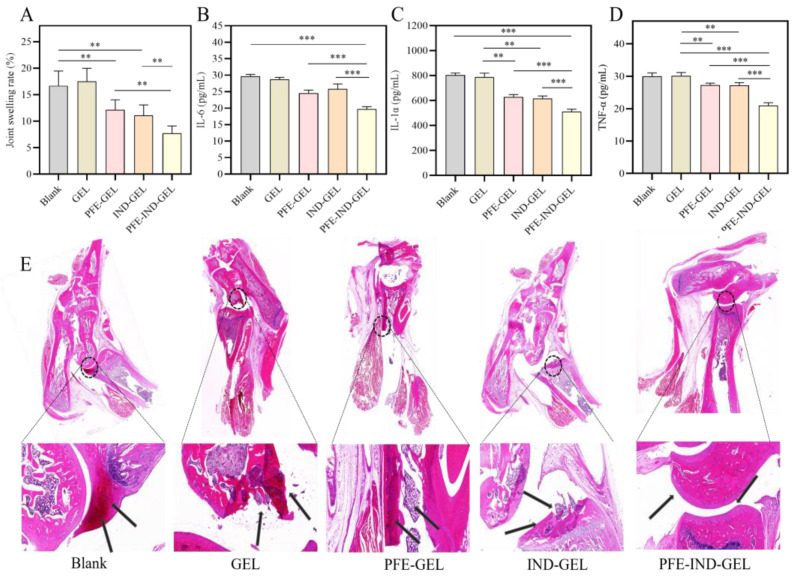
Pharmacodynamic effect of Blank, GEL, PFE-GEL, IND-GEL, and PFE-IND-GEL in IA model rats. (**A**): Rat joint swelling rate. (**B**–**D**): Inflammatory factor IL-6, IL-1α, and TNF-α. (**E**): Joint tissue section: in the blank and GEL group, obvious thickening of the synovium, proliferation of blood vessels, enlargement of the synovial cavity, and increased amount of synovial fluid could be observed; the last three treatment groups, especially the PFE-IND-GEL group, showed smooth and shiny synovium without any obvious inflammatory cell infiltration. ** *p* < 0.01; and *** *p* < 0.001.

As illustrated in Figure 4A, the joint swelling rates of rats treated with PFE-IND-GEL, PFE-GEL, and IND-GEL were significantly lower than those observed in the Blank and GEL groups. Furthermore, the joint swelling rate in the PFE-IND-GEL group was markedly reduced compared to that in both the PFE-GEL and IND-GEL groups. Figure 4B–D demonstrate that levels of inflammatory factors IL-6, IL-1α, and TNF-α in rat plasma were also significantly decreased in the treatment groups (PFE-IND-GEL, PFE-GEL, and IND-GEL) when compared to the Blank and GEL groups. Additionally, inflammatory factor levels in plasma from rats treated with PFE-IND-GEL were substantially lower than those found in both the PFE-GEL and IND-GEL groups. The histological examination of HE-stained sections from joints across different treatment groups is presented in Figure 4E. Notably, joints from rats within the Blank and GEL groups exhibited pronounced signs of arthritis characterized by inflammatory cell infiltration, synovial thickening, enlargement of the synovial cavity, and increased synovial fluid accumulation. The symptoms observed in the PFE-GEL group were somewhat milder; however, they still displayed evidence of inflammatory cell infiltration along with synovial thickening. In contrast to this finding, only minimal inflammatory cell infiltration was noted within joints from rats receiving IND-GEL treatment. Remarkably, no significant arthritic symptoms were evident among rats treated with PFE-IND-GEL; their synovia appeared smooth and glossy without any apparent abnormalities.

## 4. Discussion

The integration of botanical exosomes with advanced nanogel engineering represents a transformative approach to overcoming the dual challenges of topical permeability and sustained anti-inflammatory efficacy in IA management [16]. Notably, GelMA hydrogel serves as a promising carrier owing to its three-dimensional porous structure, excellent biocompatibility, and adjustable mechanical properties. Isik et al. [17] and Zhao et al. [18] utilized GelMA nanogel to encapsulate exosomes derived from human periodontal ligament stem cells and those obtained from human umbilical vein endothelial cells, respectively, for the treatment of inflammation. This approach offers a novel perspective on the combined therapeutic application of nanogels and exosomes in disease management.

In this study, we initially identified that PFE can enhance the penetration of the stratum corneum by inhibiting the IL-17 pathway, which plays a critical role in improving topical drug absorption [10]. The 63.2% reduction in ZO-1 mRNA expression and 62.3% decrease in membrane-localized Occludin protein surpass the 30–45% barrier-opening effects reported for gold-standard chemical enhancers like oleic acid or ethanol, while avoiding their inherent cytotoxicity [19]. This phenomenon aligns with emerging understanding of plant exosome-mediated intercellular communication, where specific miRNAs (e.g., miR-168a, miR-156) have been shown to regulate claudin family expression through Wnt/β-catenin signaling, though our transcriptomic data uniquely implicate IL-17A suppression as the primary mechanism. The observed 3.8-fold upregulation of IL-17A antagonists, coupled with 2.1-fold downregulation of FoxO1 (a known mediator of synovial hyperplasia), establishes PFE as multifunctional modulators of both epithelial barriers and inflammatory cascades—a combination absent in synthetic exosome mimetics or lipid-based carriers.

Plant-derived extracellular vesicles are increasingly recognized as promising therapeutic agents, attributed to their natural abundance and easy accessibility [14]. Huang et al. were the first to discover that PFE can inhibit the migration of neutrophils in inflammatory sites and can regulate inflammatory pathways. In this study, the in vivo pharmacodynamics experiments demonstrated that the joint swelling rate of the IA model rats in the PFE-GEL treatment group was significantly lower than that observed in both the blank control group and the GEL group. Additionally, levels of inflammatory factors exhibited a similar trend. These findings indicate the anti-inflammatory effect of PFE. However, this study does not provide conclusive evidence regarding whether PFE or IND exhibits superior anti-inflammatory effects; this limitation represents a shortcoming of our research. Furthermore, it is important to note that PFE comprises complex components, including various lipids and proteins. The specific constituents responsible for its anti-inflammatory activity require further investigation in subsequent studies. Notably, the topical products available on the market contain 1–2% and even up to 10% IND, while the nanogels prepared in this study contained only approximately 4% of IND, yet they exhibited excellent anti-IA effects.

Nanogels are innovative drug delivery systems composed of 3D networks formed by high molecular polymers and cross-linking. Their unique structure facilitates the encapsulation of anti-inflammatory drugs, allowing for sustained and targeted release to improve efficacy in the body. From the SEM images, it is evident that the prepared nanogels exhibit a characteristic 3D network structure. There are no significant differences observed in the images of PFE-GEL, IND-GEL, and PFE-IND-GEL, suggesting that neither PFE nor IND influences the internal network architecture of the nanogels, and all components are encapsulated within the nanogel matrix. The gel’s fluidity significantly impacts its skin adhesion. The nanogel in this study exhibited high fluidity before application, but after 2 min of ultraviolet exposure, its fluidity decreased notably. This reduction is essential for improving skin adhesion.

Overall, the excellent anti-IA effect of PFE-IND-GEL can be attributed to the following three points: (1) Compared to the oral administration route, the local skin penetration administration route for arthritis not only significantly reduces the distance from the site of administration to the absorption vessels but also circumvents the complex environment of the gastrointestinal tract [20]. (2) PFE decreases the expression of tight junction proteins in epidermal keratinocytes, thereby enhancing permeability and facilitating the absorption of IND. (3) PFE is derived from *Perilla frutescens* leaves and may possess certain anti-inflammatory properties [21]. While this study establishes proof-of-concept for plant exosome-enabled topical therapy, several translational challenges require attention.

## 5. Conclusions

This study presents a PFE-based topical drug delivery nanogel that exhibits dual efficacy by enhancing skin permeation and functioning as an effective anti-inflammatory agent. The system’s ability to modulate epithelial barrier integrity while providing sustained drug release offers a transformative strategy for inflammatory arthritis management, bridging botanical therapeutics and advanced drug delivery technologies. This approach establishes a foundation for developing nature-inspired, precision-targeted therapies for chronic inflammatory disorders.

## Figures and Tables

**Table 1:** The exact composition of the GEL.

Formulations	Oxidized Alginate	GelMA	LAP	CaCl_2_	PFE	IND	Water
PFE-GEL	3.75 mL	5 mL	0.25 mL	5 mL	1 mL		10 mL
IND-GEL	3.75 mL	5 mL	0.25 mL	5 mL		1 g	11 mL
PFE-IND-GEL	3.75 mL	5 mL	0.25 mL	5 mL	1 mL	1 g	10 mL

**Table 2:** Primer sequences for qPCR analysis.

Target	Forward Primer (5′-3′)	Reverse Primer (5′-3′)
ZO-1	CGGAAAGGGTGAAGGAGATG	CATTGCCATCCTCTTCATCC
Occludin	TGTGGGTTCCTACTGCTGCT	AGCCACAGCCATTGTAGTCC
GAPDH	GGAGCGAGATCCCTCCAAAAT	GGCTGTTGTCATACTTCTCATGG

## Data Availability

Data are contained within the article.

## Abbreviations

The following abbreviations are used in this manuscript:

IA	Inflammatory arthritis
IND	Indomethacin
PFE	Perilla Frutescens leaf-derived exosome-like nanovesicles
PFE-IND-GEL	PFE loaded with IND and developed into a nanogel designed
TDDS	Topical drug delivery systems
TEM	Transmission electron microscopy

## References

[1] Wedderburn L.R., Ramanan A.V., Croft A.P., Hyrich K.L., Dick A.D. (2023). Towards molecular-pathology informed clinical trials in childhood arthritis to achieve precision medicine in juvenile idiopathic arthritis. Ann. Rheum. Dis..

[2] Madsen C.M., Primdahl J., Bremander A., Eggen L., Christensen J.R. (2023). Developing a complex vocational rehabilitation intervention for patients with inflammatory arthritis: The WORK-ON study. BMC Health Serv. Res..

[3] Ursum J., Nielen M.M., Twisk J.W., Peters M.J., Schellevis F.G., Nurmohamed M.T., Korevaar J.C. (2013). Increased risk for chronic comorbid disorders in patients with inflammatory arthritis: A population-based study. BMC Fam. Pract..

[4] Li W., Yu L., Li W., Ge G., Ma Y., Xiao L., Qiao Y., Huang W., Huang W., Wei M. (2023). Prevention and treatment of inflammatory arthritis with traditional Chinese medicine: Underlying mechanisms based on cell and molecular targets. Ageing Res. Rev..

[5] Bañuls-Mirete M., Ogdie A., Guma M. (2020). Micronutrients: Essential Treatment for Inflammatory Arthritis?. Curr. Rheumatol. Rep..

[6] Hosseinikhah S.M., Barani M., Rahdar A., Madry H., Arshad R., Mohammadzadeh V., Cucchiarini M. (2021). Nanomaterials for the Diagnosis and Treatment of Inflammatory Arthritis. Int. J. Mol. Sci..

[7] Katz J.N., Arant K.R., Loeser R.F. (2021). Diagnosis and Treatment of Hip and Knee Osteoarthritis: A Review. JAMA.

[8] Elmunzer B.J., Hernandez I., Gellad W.F. (2020). The Skyrocketing Cost of Rectal Indomethacin. JAMA Intern. Med..

[9] Samy S., Shehata M., Albuhiri A., Khairy A. (2022). Combined rectal indomethacin and intravenous saline hydration in post-ERCP pancreatitis prophylaxis. Arab J. Gastroenterol..

[10] Lim D., Song M., Kim M., Park H.K., Kim D.W., Pang C. (2025). Bioinspired Suction-Driven Strategies with Nanoscale Skin-Controllable Adhesive Architectures for Efficient Liquid Formulated Transdermal Patches. ACS Nano.

[11] Zhang Y., Zhao B., Wang J., Zhang Z., Shen M., Ren C., Li M., Liu M., You Z., Li P. (2025). Therapeutic potential of Lycium barbarum-derived exosome-like nanovesicles in combating photodamage and enhancing skin barrier repair. Extracell. Vesicle.

[12] Liu D., Wang Y., Lu Z., Lv F., Bie X., Zhao H. (2022). Separation, characterization and anti-inflammatory activities of galactoglycerolipids from *Perilla frutescens* (L.) Britton. Nat. Prod. Res..

[13] Liu B.H., Li Z.H., Wang B.R., Zhou J., Zhang B., Wang K.L., Zhang Y.H., Mu Z.S. (2025). Rosmarinic acid in *Perilla frutescens* L. as a potential adenosine deaminase inhibitor: Preparation, machine learning validation and binding mechanism study. Food Chem..

[14] Huang J., Chen L., Li W., Chang C.J. (2025). Anti-inflammatory and antioxidative effects of Perilla frutescens-derived extracellular vesicles: Insights from Zebrafish models. Mol. Immunol..

[15] Li P., Lu M., Peng T., Wu Y., Zhu L., Liu Y., Zhang W., Xiang T. (2024). An improvised one-step OptiPrep cushion ultracentrifugation method for outer membrane vesicles isolation of Klebsiella pneumoniae. BMC Microbiol..

[16] Li Y., Zhu Z., Li S., Xie X., Qin L., Zhang Q., Yang Y., Wang T., Zhang Y. (2024). Exosomes: Compositions, biogenesis, and mechanisms in diabetic wound healing. J. Nanobiotechnology.

[17] Isik M., Vargel I., Ozgur E., Cam S.B., Korkusuz P., Emregul E. (2023). Human periodontal ligament stem cells-derived exosomes-loaded hybrid hydrogel enhances the calvarial defect regeneration in middle-age rats. Mater. Today Commun..

[18] Zhao D., Yu Z., Li Y., Wang Y., Li Q., Han D. (2020). GelMA combined with sustained release of HUVECs derived exosomes for promoting cutaneous wound healing and facilitating skin regeneration. J. Mol. Histol..

[19] Bi H., Wang F., Lin L., Zhang D., Chen M., Shang Y., Hua L., Chen H., Wu B., Peng Z. (2025). The T-type voltage-gated Ca(2+) channel Ca(V)3.1 involves in the disruption of respiratory epithelial barrier induced by Pasteurella multocida toxin. Virulence.

[20] Manara M., Carriero A., Congia M., Galluzzo C., Pandolfi M., Salvato M., Scriffignano S., Cauli A., D’Angelo S., Lubrano E. (2025). Non-steroidal anti-inflammatory drugs in psoriatic arthritis: Clinical practice suggestions based on scientific evidence and expert opinion. Clin. Exp. Rheumatol..

[21] Wang Z., Wang D., Fang J., Song Z., Geng J., Zhao J., Fang Y., Wang C., Li M. (2024). Green and efficient extraction of flavonoids from *Perilla frutescens* (L.) Britt. leaves based on natural deep eutectic solvents: Process optimization, component identification, and biological activity. Food Chem..

